# Rural–Urban Differences in the Utilization of Hospital-Based Care for Women of Reproductive Age

**DOI:** 10.1089/whr.2021.0061

**Published:** 2022-01-31

**Authors:** Ching-Ching Claire Lin, Hyunjung Lee, John E. Snyder

**Affiliations:** ^1^ Institute of Health Policy and Management, College of Public Health, National Taiwan University, Taipei, Taiwan. Formally Office of Planning, Analysis, and Evaluation (OPAE), Health Resources and Services Administration (HRSA), U.S. Department of Health and Human Services (HHS), Rockville, Maryland, USA.; ^2^ Oak Ridge Institute for Science and Education (ORISE), Oak Ridge, Tennessee, USA.; ^3^ Office of Health Equity (OHE), Health Resources and Services Administration (HRSA), U.S. Department of Health and Human Services (HHS), Rockville, Maryland, USA.; ^4^ Office of Planning, Analysis, and Evaluation (OPAE), Health Resources and Services Administration (HRSA), U.S. Department of Health and Human Services (HHS), Rockville, Maryland, USA.

**Keywords:** Rural health, women of reproductive age, hospital-based outpatient, emergency department visit, hospitalization, health insurance

## Abstract

***Background:*** To investigate rural–urban differences in hospital-based care utilization among women of reproductive age (18–44 years).

***Methods:*** Rural–urban differences were estimated for hospital outpatient visits, emergency department (ED) visits, hospitalizations, and associated expenditures both overall and by insurance status, by analyzing a nationally representative sample of women of reproductive age from the Medical Expenditure Panel Survey (2006–2015).

***Results:*** The study sample consisted of 48,114 women of reproductive age. Unadjusted results showed that rural women reported higher likelihood of hospital outpatient visits (rural vs. urban: 17.10% vs. 13.34%) although, among those using such care, fewer average visits (rural vs. urban: 2.00 vs. 2.56 visits). Rural women reported higher likelihood of ED visits (rural vs. urban: 18.13% vs. 15.11%) and more hospital stays (rural vs. urban: 0.13 vs. 0.11 stays). Adjusted results showed rural women had higher likelihood of outpatient care use (+2.5 percentage points; 95% confidence interval [CI] = 0.002–0.049) but fewer visits (−0.314 visits, 95% CI = −0.566 to −0.062). For the privately insured, rural women had greater likelihood of outpatient care (+3.1 percentage points, 95% CI = 0.001–0.060) and fewer ED visits (−0.031 visits, 95% CI = −0.061 to −0.003); for the publicly insured, rural women had more hospital stays (+0.045 stays, 95% CI = 0.009–0.083); for the uninsured, rural women had fewer outpatient visits among those using such care (−1.118 visits, 95% CI = −1.865 to −0.372) and shorter hospital stays overall (−0.093 nights, 95% CI = −0.181 to −0.005). Rural–urban expenditure differences were not significant between any insurance grouping.

***Conclusions:*** Rural–urban differences in hospital-based care utilization were observed, although somewhat heterogeneous by insurance status. Strengthening outpatient and preventive service access, particularly for publicly insured and uninsured rural women of reproductive age, is important for shifting care to lower cost settings and improving population health.

## Introduction

Costs related to pregnancy and postpartum care in the United States amounted to roughly $71.3 billion in 2016, and comprised one of the highest condition-related categories of national health care spending.^1^ Despite considerable and escalating national health care spending on the delivery of care to women of reproductive age, rates of severe maternal morbidity and mortality are rising in the United States and exceed those of most other high-income countries.^2–5^ This growing mismatch between resource utilization and outcomes clearly indicates a need for further investigation, especially when considering that a substantial portion of maternal deaths is likely preventable.^6–8^

Calls to action have cited a need for a comprehensive exploration of outcomes determinants for women of reproductive age, including the roles of geography and rurality, the utilization and quality of existing pre- and perinatal health care resources, hospital-based care, and other factors.^9,10^ Geographic location, and rurality in particular, appears to affect both access to care and birth outcomes for women of reproductive age.^9–16^ Although maternal deaths and deliveries requiring emergency, life-saving treatment appear to be increasing in both rural and urban areas, rural women face greater risks of having adverse childbirth events.^15^

The preventability of many maternal deaths implies a necessity to both strengthen prenatal and preventive care delivery in the outpatient setting and also to improve the timeliness, breadth, and quality of hospital-based care provided to women, particularly in rural areas.^9^ Yet, counteracting this dual need, women from rural areas have been reported to be more likely to delay or forgo seeking needed health care.^16^ Rural care access and utilization disparities have been attributed to a variety of factors, including higher rates of poverty and uninsurance, greater Medicaid reliance, less robust provider availability, longer travel distances to health care facilities, declining rural obstetric service access, and rising rural hospital closure rates.^14,16–21^

In addition, insurance status appears to play a particularly critical role in determining access to and availability of care in rural areas.^22^ Women of reproductive age with public sources of health insurance tend to receive preventive care at lower rates than those who are privately insured, and they are also more likely to access primary care services through emergency departments (EDs).^23^ Although a wide range of prior studies have investigated women's health service use both at hospitals and in outpatient settings, and the effects of insurance status and rural residence on care utilization, none have explored the intersection of these factors for women during the peak ages of fertility.^10–12^^,^^24–32^

As such, this investigation aims to examine how rurality and insurance status affect the utilization of and expenditures for hospital-based outpatient, ED, and inpatient care by women of reproductive age.

## Materials and Methods

### Data and study sample

The main dataset for this study was 10 years (2006–2015) of pooled Medical Expenditure Panel Survey (MEPS) data from the Agency for Healthcare Research and Quality (AHRQ). MEPS is a set of large-scale, nationally representative surveys, and is considered the nation's most complete source of data on the cost and utilization of health care services.^33^

To define rural–urban residence for respondents, MEPS data were matched with Rural-Urban Commuting Area (RUCA) codes, which use commuting pattern information to characterize both rural status and rural–urban relationships within the nation's census tracts. Consistent with Health Resources and Services Administration (HRSA) policy for defining rurality, geographical tracts with RUCA codes between 4 and 10 were considered rural in this study. In addition, the MEPS dataset was merged with HRSA's Area Health Resource Files (AHRF) for the study of relevant county-level health care access variables. The AHRF include a wide array of health care and population data from numerous sources at the county, state, and national levels.^34^

The study sample included all women of reproductive age during the study period, defined here as 18–44 years old. This age group encompasses the main users of maternal health care services and accounted for 99% of births in 2018.^35^ Accounting for missing values for rural status and/or covariates among MEPS respondents where present (18,318; see Appendix Table A1), the final pooled sample size was 48,114. Consistent with other recent investigations, the focus of this study was on all women in the peak ages of fertility seeking hospital-based care, and not solely women seeking care specifically for obstetrical delivery.^10–13^

This approach is inclusive of health care provided to women before, contemplating, during, and after pregnancy, as well as individuals who never became pregnant. Our analytic sample, therefore, captured hospital-based care related to the management of acute and chronic diseases associated with higher perinatal health risks such as asthma and diabetes, obstetrical care, and postpartum care delivered within 42 days of childbirth or termination of pregnancy. The latter is consistent with the Centers for Disease Control and Prevention (CDC) definition of a maternal mortality event.^36,37^

This study compared utilization and costs related to outpatient, ED, and inpatient hospital care use between rural and urban residents among all women of reproductive age and by their insurance status. Individuals were further categorized into three different insurance categories representing their status during the preceding year. The privately insured group included persons with private insurance coverage or TRICARE (formally known as Civilian Health and Medical Program of the Uniformed Services, CHAMPVA), at any time during the year. The publicly insured group included persons with only public insurance coverage during a year, including Medicaid or State Children's Health Insurance Program (SCHIP), Medicare, and other public hospital and physician coverage. The uninsured group are persons who were uninsured during the entire year.

### Measures

The key outcomes of interest around hospital utilization included hospital-based outpatient visits, ED visits, inpatient stays, and total hospital charges. Probabilities of using a specific type of service and level of utilization for each type of services were assessed separately. For probabilities of using a specific type of service, three binary outcomes were defined as whether an individual reporting having had at least one visit or encounter in the preceding year (yes = 1) to the following services: (1) any outpatient visits to a hospital outpatient department or specialty clinic; (2) any ED visits; and (3) any inpatient hospitalization stays.

Then, levels of hospital-based care utilization were estimated as the total numbers of outpatient visits, ED visits, inpatient stays, and nights of inpatient hospitalization. Inpatient stays of <24 hours were counted as one hospital night. For a more robust measurement of utilization levels, number of counts/nights were estimated for all individuals in the study sample as well as for restricted samples of individuals with at least one encounter for each type of service. Finally, total expenditures for each type of hospital-based care event were estimated, defined as the summation of facility and physician charges in MEPS.

Using earlier studies on women's health care access and health disparities for guidance, the current analyses were adjusted for a number of individual-level and county-level factors that may impact both a person's health care use and selection into rural residence.^11–13,16,18,21,38^ For example, poverty, employment status, and educational achievement levels can all influence both where one lives, one's access to care, and one's health outcomes.^39^ Individual-level covariates examined in this study included age, race, ethnicity, educational status, marital status, family size, employment status and family income, pregnancy status, U.S. Census region, self-perceived poor or fair general and mental health status, and the number of chronic disease diagnoses.

As defined by MEPS, the number of chronic diseases included counts for the following conditions reported by the respondents: diabetes, lung disease (emphysema or asthma), heart disease (coronary disease, angina, heart attack, other heart disease), stroke, and high blood pressure. County-level factors included county unemployment rates (calculated as the ratio of unemployed individuals to the civilian labor force times 100), the density of available primary care physicians (the number of family physicians, general practice, and internal medicine physicians per 1,000 total population), and available hospital bed density (number of hospital beds per 1,000 total population). The first covariate was a proxy for job opportunities in a community, where the latter two covariates were used as a general proxy for location-specific access to health care.

### Analyses

Descriptive analyses were conducted to compare unadjusted differences, by rural residence, for all types of hospital-based care utilization and associated expenditures. Various regression models were used to compare adjusted differences between rural and urban residents on health care utilization and expenditures. For probabilities of using a specific type of service, a logit regression model was applied and differential effects of rural residency were estimated. For estimation for each level of outpatient, ED, and inpatient utilization, a generalized linear model was used with negative-binomial family and log link. This estimation for utilization level was also conducted on a subsample of actual utilizers using each specific type of care. For example, analyses on the level of outpatient care (number of visits) were conducted on all women as well as those with at least one outpatient visit during the year (utilizers).

For expenditure analyses, a generalized linear model with gamma family and log link was estimated for outpatient care expenditure, ED care discharges, and inpatient care discharges. All analyses were conducted on a full sample of women of reproductive age as well within three subgroups based on insurance status (privately insured, publicly insured, and uninsured). Furthermore, complex survey design was used in all adjusted and unadjusted analyses, accounting for clustering, multiple stages of selection, and disproportionate sampling. All analyses were conducted using Stata, version 15. Differential effects of rural residence were computed with delta-methods standard errors. This study was deemed exempt from Institutional Review Board review as it utilized de-identified, publicly available, population-level datasets.

## Results

### Study sample

Table 1 presents a descriptive summary of the total study sample and across the three insurance subgroups examined, stratified by rural/urban residence. The average age of all women of reproductive age in the study was 31 years old, and 12% were pregnant. The majority of women were non-Hispanic white (59%), and most held a bachelor's degree or higher (50%). Forty-five percent of the study sample were married, with an average family size of 3.2 persons. The majority were employed (61%). Slightly over one third (36%) lived in low-income families; 31% lived in middle-income and 33% in high-income families. As a whole, most women reported overall good health, although 13% reported activity limitations, 7% reported fair or poor health, 4% reported fair or poor mental health, and roughly one quarter reported having some chronic conditions. Geographically, 34% of women lived in the South, 25% in the West, 22% in the Midwest, and 19% in the Northeast.

**Table 1.** Selected Characteristics of the Women of Reproductive Age in the Study, 2006–2015

	Study population			Privately insured^a^			Publicly insured			Uninsured		
	All	Rural	Urban	All	Rural	Urban	All	Rural	Urban	All	Rural	Urban
Sample size (*n*)	48,114	4,082	44,032	26,668	2,029	24,639	11,265	1,035	10,230	10,181	1,018	9,163
Individual-level characteristics												
Age, years	31.02	30.79	31.05	31.52	31.31	31.54	29.25	29.24	29.26	30.61	30.70	30.60
Race and ethnicity												
Non-Hispanic White, %	58.68	74.10	57.12	66.36	80.76	65.05	41.76	66.62	38.62	41.65	58.91	39.58
Black, %	14.11	10.07	14.52	11.57	7.51	11.94	24.99	15.10	26.24	14.17	13.54	14.25
Hispanic, %	18.38	10.13	19.21	12.57	7.18	13.06	25.87	10.53	27.81	36.89	20.10	38.90
Other, %	8.83	5.70	9.15	9.51	4.56	9.96	7.39	7.74	7.34	7.29	7.44	7.27
Education level												
Less than high school, %	14.60	17.91	14.27	8.12	10.33	7.92	32.46	32.05	32.51	25.18	28.96	24.72
High school, %	35.15	41.62	34.50	30.71	39.06	29.96	44.36	48.76	43.81	45.59	42.73	45.93
Bachelor's degree/graduate school, %	50.25	40.47	51.23	61.17	50.60	62.13	23.18	19.19	23.68	29.23	28.31	29.34
Marital/family status												
Married, %^b^	45.45	49.88	45.01	51.92	58.01	51.37	24.30	29.94	23.59	38.39	43.29	37.81
Family size, number of persons	3.29	3.46	3.27	3.17	3.40	3.14	3.62	3.57	3.62	3.51	3.55	3.50
Employment, %	60.68	56.58	61.09	70.92	69.25	71.07	29.11	26.16	29.48	47.49	45.55	47.72
Family income^c^												
Low family income, %	35.69	46.02	34.65	18.85	27.39	18.08	81.41	82.78	81.24	63.91	70.97	63.06
Middle family income, %	31.15	34.25	30.84	36.14	43.07	35.51	14.54	14.57	14.54	26.07	24.94	26.20
High family income, %	33.15	19.73	34.50	45.01	29.54	46.41	4.05	2.64	4.23	10.02	4.09	10.74
Health status												
Activity limitation, %	12.89	14.72	12.70	10.01	10.56	9.96	26.33	29.26	25.96	11.77	13.30	11.59
Poor or fair health status, %	6.80	10.19	6.46	4.30	6.65	4.09	16.56	20.58	16.05	7.87	11.19	7.47
Poor or fair mental health status, %	4.43	5.80	4.29	2.88	3.57	2.82	11.65	12.74	11.51	3.84	5.95	3.59
Pregnancy status, %	12.46	13.33	12.38	11.73	11.57	11.74	21.38	23.66	21.10	6.38	8.12	6.18
Chronic disease diagnoses, %												
0^d^	75.09	72.16	75.39	76.26	74.12	76.45	66.52	63.41	66.91	78.88	74.92	79.35
1	19.81	20.07	19.78	19.59	19.84	19.56	23.36	21.49	23.60	17.07	19.28	16.81
2	4.18	6.21	3.97	3.63	5.00	3.51	7.31	11.41	6.79	3.35	4.73	3.19
3+	0.92	1.56	0.85	0.53	1.03	0.48	2.80	3.69	2.69	0.69	1.07	0.65
Region												
Northeast	19.49	13.87	20.06	20.02	15.05	20.47	24.20	13.84	25.50	12.15	9.75	12.44
Midwest	21.69	30.89	20.76	22.89	34.72	21.82	22.07	24.58	21.75	15.80	24.36	14.78
South	34.29	34.60	34.26	33.27	31.79	33.41	27.27	34.19	26.39	46.33	44.93	46.50
West	24.53	20.65	24.92	23.82	18.44	24.30	26.47	27.39	26.35	25.72	20.96	26.29
County-level characteristics												
Unemployment rate^e^	6.88	7.34	6.83	6.67	7.09	6.64	7.44	8.08	7.36	7.20	7.40	7.18
Number of hospital beds per 1,000 population	3.22	3.63	3.18	3.22	3.77	3.17	3.27	3.36	3.26	3.17	3.43	3.14
Number of primary physicians per 1,000 population (2010)^f^	0.67	0.46	0.69	0.68	0.47	0.70	0.66	0.44	0.69	0.60	0.41	0.62

^a^ Privately insured: Persons with private insurance coverage or TRICARE/CHAMPVA at any time during the year of study. Publicly insured: Persons with only public insurance coverage during a year, including Medicaid or SCHIP, Medicare, and other public insurance types. Uninsured: Persons uninsured during the entire year.

^b^ Marital status is a binary measure (Yes = married; No = widowed/divorced/separated/never married).

^c^ Low income is defined in MEPS as a family income <200% of the poverty line, middle income as 200% to <400% of the poverty line, and high income as ≥400% of the poverty line.

^d^ Number of chronic diseases present, as self-identified by MEPS respondents, among the following conditions: diabetes, lung disease (emphysema or asthma), heart disease (coronary, angina, heart attack, other heart disease), stroke, high blood pressure.

^e^ The calculated county unemployment rate is the ratio of unemployed to the civilian labor force [(unemployed/labor force) × 100].

^f^ Primary care physician density is defined as the number of family physicians, general practice, and internal medicine physicians per 1,000 total population.

MEPS, Medical Expenditure Panel Survey; SCHIP, State Children's Health Insurance Program.

Most women in the study were privately insured (*n* = 26,668), followed by those with public insurance (*n* = 11,265) and individuals who were uninsured (*n* = 10,181). There were some observed demographic variations across different insurance groups. Among privately insured women, the majority were non-Hispanic white (66%), employed (71%), married (52%), and held a bachelor's degree or higher (61%); 45% reported high family income. Black women were most represented in the publicly insured group (25%), and Hispanic women in the uninsured group (37%). Attainment of a high-school level education was observed at the highest proportions in publicly insured (44%) and uninsured groups (46%), as was low family income (81% and 64%, respectively). Pregnancy was most commonly reported among the publicly insured (21%). Summary statistics were also stratified by rural–urban status among all as well as within each insurance status group.

### Utilization

The unadjusted analysis of utilization of hospital-based care (Table 2) showed that, among all women of reproductive age in the study, those living in rural areas were more likely to have had hospital outpatient department visits (rural vs. urban: 17.10% vs. 13.34%) although, for those with at least one visit, fewer visits on average (rural vs. urban: 2.00 vs. 2.56 visits). Living in a rural area was associated with a higher likelihood of ED visits (rural vs. urban: 18.13% vs. 15.11%) and a higher number of hospitalizations (rural vs. urban: 0.13 vs. 0.11 hospital stays).

**Table 2.** Unadjusted Annual Health Care Utilization Among Women of Reproductive Age, 2006–2015

	Sample size	All		Privately insured^a^		Publicly insured		Uninsured	
		Rural	Urban	Rural	Urban	Rural	Urban	Rural	Urban
Hospital-based outpatient visits									
Any visits	48,107	17.10%^*^	13.34%^*^	19.43%^*^	14.44%^*^	18.65%	16.09%	7.18%	5.35%
Number of visits	48,107	0.34	0.34	0.38	0.34	0.43	0.55	0.12	0.13
Number of visits (nonzero group only)	5,679	2.00^*^	2.56^*^	1.94^*^	2.35^*^	2.30	3.40	1.61	2.44
ED visits									
Any visits	48,112	18.13%^*^	15.11%^*^	13.34%	12.32%	32.17%	28.52%	19.48%	14.02%
Number of visits	48,112	0.27	0.22	0.18	0.17	0.52	0.49	0.30	0.20
Number of visits (nonzero group only)	7,774	1.47	1.48	1.33	1.37	1.62	1.71	1.54	1.44
Hospitalizations									
Any stays	48,114	10.61%	9.12%	8.98%	8.33%	22.03%	17.30%	3.74%	4.18%
Number of inpatient hospitalizations	48,114	0.13^*^	0.11^*^	0.10	0.10	0.29^*^	0.21^*^	0.04	0.04
Number of nights in the hospital	48,114	0.43	0.34	0.26	0.28	1.29	0.80	0.11	0.15
Number of inpatient hospitalizations (nonzero group only)	4,471	1.20	1.15	1.11	1.14	1.33	1.20	1.15	1.06
Number of nights in the hospital (nonzero group only)	4,471	4.11	3.77	2.89	3.39	5.86	4.64	3.01	3.50
Expenditures (USD)									
Hospital-based outpatient visits	48,114	$301.90	$335.98	$396.37	$400.51	$181.23	$305.27	$102.46	$67.10
ED visits	48,114	198.55	191.49	196.60	193.01	238.02	234.63	161.73	139.05
Hospitalizations	48,114	1,058.66	980.99	1,013.40	1,024.93	1,977.85	1,478.35	200.99	253.32

^*^ *p* < 0.05.

^a^ Privately insured: Persons with private insurance coverage or TRICARE/CHAMPVA, at any time during the year of study. Publicly insured: Persons with only public insurance coverage during a year, including Medicaid or SCHIP, Medicare, and other public insurance types. Uninsured: Persons uninsured during the entire year.

ED, emergency department.

When rural–urban differences were assessed within each insurance status group, three results in particular stand out. Rural women of reproductive age with private insurance were more likely to have had hospital outpatient department visits (rural vs. urban: 19.43% vs. 14.44%), although fewer of them on average (rural vs. urban: 1.94 vs. 2.35 visits) among those with at least one visit. Rural residents with public insurance had higher average numbers of hospitalizations (rural vs. urban: 0.29 vs. 0.21 hospital stays). Unadjusted rural–urban differences in hospital-based care utilization did not reach a level of statistical significance among women without health insurance.

After adjusting for covariates that may impact health care use and rural residence, a few findings come to light (Table 3). For one, among all women of reproductive age in our study sample, those living in rural areas were more likely than urban women to have utilized hospital outpatient departments by 2.5 percentage points (95% confidence interval [CI]: 0.002–0.049), although the average number of visits among rural women who utilized such care is marginally lower by −0.314 visits (95% CI: −0.566 to −0.062) than it is for urban counterparts.

**Table 3.** Differential Differences (Adjusted) of Rural Residency on Annual Hospital-Based Health Care Utilization Among Women of Reproductive Age, 2006–2015

	Sample size	All (95% CI)	Privately insured^a^ (95% CI)	Publicly insured (95% CI)	Uninsured (95% CI)
Hospital-based outpatient visits					
Any visits	48,107	0.025^*^ (0.002 to 0.049)	0.031^*^ (0.001 to 0.060)	0.022 (−0.021 to 0.066)	0.010 (−0.012 to 0.032)
Number of visits	48,107	0.015 (−0.050 to 0.079)	0.039 (−0.033 to 0.111)	−0.004 (−0.190 to 0.182)	−0.032 (−0.107 to 0.042)
Number of visits (nonzero group only)	5,679	−0.314^*^ (−0.566 to −0.062)	−0.189 (0.493 to 0.115)	−0.245 (−0.729 to 0.240)	−1.118^**^ (−1.865 to −0.372)
ED visits					
Any visits	48,112	−0.002 (−0.017 to 0.013)	−0.012 (−0.027 to 0.004)	−0.0002 (−0.0418 to 0.0412)	0.018 (−0.015 to 0.051)
Number of visits	48,112	−0.016 (−0.044 to 0.013)	−0.031^*^ (−0.061 to −0.003)	−0.026 (−0.107 to 0.055)	0.028 (−0.032 to 0.088)
Number of visits (nonzero group only)	7,774	−0.079 (−0.162 to 0.003)	−0.126^*^ (−0.240 to −0.013)	−0.095 (−0.247 to 0.057)	0.021 (−0.148 to 0.191)
Hospitalizations					
Any stays	48,114	0.0002 (−0.008 to 0.009)	−0.002 (−0.015 to 0.010)	0.018 (−0.003 to 0.040)	−0.0162 (−0.0326 to 0.0002)
Number of inpatient hospitalizations	48,114	0.001 (−0.012 to 0.015)	−0.011 (−0.028 to 0.006)	0.045^*^ (0.009 to 0.083)	−0.015 (−0.033 to 0.003)
Number of nights in the hospital	48,114	−0.045 (−0.134 to 0.044)	−0.076 (−0.160 to 0.007)	0.135 (−0.150 to 0.420)	−0.093^*^ (−0.181 to −0.005)
Number of inpatient hospitalizations (nonzero group only)	4,471	0.031 (−0.031 to 0.093)	−0.049 (−0.117 to 0.018)	0.108^*^ (0.012 to 0.205)	0.107 (−0.018 to 0.232)
Number of nights in the hospital (nonzero group only)	4,471	−0.003 (−0.470 to 0.464)	−0.306 (−0.799 to 0.187)	0.405 (−0.502 to 1.312)	0.104 (−0.885 to 0.677)
Expenditures (USD)					
Hospital-based outpatient visits	48,114	$103.357 (−27.533 to 234.248)	$89.631 (−50.546 to 229.807)	−$41.147 (−144.162 to 61.867)	$38.062 (−37.086 to 113.210)
ED visits	48,114	−0.900 (−55.610 to 37.610)	−11.430 (−78.404 to 55.545)	−25.941 (−81.100 to 29.217)	12.751 (−65.483 to 90.986)
Hospitalizations	48,114	443.698 (−208.578 to 1,095.974)	470.546 (−316.566 to 1,257.658)	1,066.897 (−22.259 to 2,156.053)	9,592.852 (−17,561.160 to 36,746.870)

^*^ *p* < 0.05, ^**^p < 0.01. All binary outcomes were estimated with logistic regression, all count outcomes were estimated with a generalized linear model with negative binomial family and log link, and all expenditure outcomes were estimated with a generalized linear model with gamma family and log link.

^a^ Privately insured: Persons with private insurance coverage or TRICARE/CHAMPVA at any time during the year of study. Publicly insured: Persons with only public insurance coverage during a year, including Medicaid or SCHIP, Medicare, and other public insurance types. Uninsured: Persons uninsured during the entire year.

CI, confidence interval.

The adjusted rural–urban differences in utilization were further examined by insurance status groupings. For women with private insurance, rurality is associated with higher likelihood of utilizing hospital-based outpatient care by 3.1 percentage points (95% CI: 0.001–0.060). Furthermore, for women privately insured, rurality is associated with 0.031 fewer ED visits for the whole group (95% CI: −0.061 to −0.003), and associated with 0.126 fewer visits among those with at least one ED visit (95% CI: −0.240 to −0.013). For women with public sources of insurance, rurality appears to be associated with 0.045 more hospital stays among the whole group (95% CI = 0.009–0.083) and 0.108 more stays for those who actually utilized such care (95% CI = 0.012–0.205). Finally, among uninsured rural women, rurality appears to be associated with 1.118 fewer outpatient visits among those who utilized such care (−1.118; 95% CI = −1.865 to −0.372) and with 0.093 fewer nights in the hospital (95% CI = −0.181 to −0.005).

### Expenditures

The unadjusted analysis of expenditures for hospital-based care showed that, among women of reproductive age, the average expenditures were lower for rural women for hospital outpatient department visits than for urban counterparts (rural vs. urban: $301.90 vs. $335.98). The average expenditures, however, were higher for ED visits (rural vs. urban: $198.55 vs. $191.49) and inpatient stays (rural vs. urban: $1,058.66 vs. $980.99), although none of these differences (expenditures for outpatient, ED, and inpatient care) were statistically significant (Table 2). When examining within each health insurance subgroup, rural–urban differences in expenditures for hospital outpatient care, for ED care, and for inpatient care were generally similar to what were observed for the overall group as a whole. The adjusted effects of rurality on hospital-based care expenditures (Table 3) did not reach a level of statistical significance for the study population as a whole or when analyzed in insurance-based subgroups.

## Discussion

This study serves as a starting point in comparing levels of hospital-based care utilization among women of reproductive age residing in rural and urban areas and by insurance status. The findings contribute to the growing literature on the role of rural residency and insurance coverage on access to care and health resource use. The results indicate that, during a recent 10-year period, apparent differences in hospital-based health care use between rural and urban women of reproductive age were heterogeneous by health insurance status. The greater use of ED visits, fewer numbers of outpatient visits, and higher average numbers of hospitalizations for rural women of reproductive age emphasizes the need for this population to be able to access the routine and preventive care it needs. This is essential to reduce the pregnancy-related health risks in this population, and particularly for those with public sources of or no health insurance. In considering the complex array of interactions observed between rurality, insurance, and patterns of care, three main findings have emerged in this study.

First, rural women who have public forms of insurance demonstrated higher inpatient care utilization than their urban counterparts. This finding potentially relates to insufficient rural access to the types of primary and preventive care services that can mitigate hospitalization risks among beneficiaries of public insurances, as prior literature has suggested.^16,23^ Whereas rural women more commonly delay or forgo seeking preventive health care, women of reproductive age with public sources of health insurance tend to receive less preventive care than privately insured women.^16,23^ Broadly speaking, access to recommended care and preventive services in rural communities is often also constrained by factors such as a lower supply of health care providers, more limited service availability, and unique aspects of rural culture, such as patient knowledge about and perceived value of preventive care.^12,14–21,40,41^ Barriers to preventive care increase the risks for women to develop chronic illness and complicate the ability to manage existing illness, hence driving care more toward the inpatient setting.^42^

Second, among those without insurance coverage, on average, rural women had fewer hospital-based outpatient visits among those using such care and spent fewer days in the hospital than urban women. This could suggest that uninsured women residing in rural area had a lower need for inpatient care or that they faced greater barriers to accessing hospital care overall. Given that all estimations in this study were adjusted for health status and the presence of chronic conditions, the latter possibility may be more likely. Access to hospital care in rural areas, in general, can be more difficult because of a variety of factors, such as the increasing rate of rural hospital closures observed during the past several years, closures specifically of rural obstetric units, and an overall lower supply of health care providers.^15,43–46^

Third, it is interesting to observe that privately insured women residing in rural areas were more likely to visit a hospital-based outpatient clinic and had fewer ED visits than their urban counterparts. Such findings could be interpreted to suggest that, when they have private health insurance, rural women could have equivalent or better access to hospital-based outpatient care than urban women. If this is the case, owing to factors such as higher rural availability levels of physician assistants and advance practice nurses as one example, this would be an interesting area for further investigation.^47^ Outpatient care settings provide the greatest opportunity for obstetrician-gynecologists and primary care providers to provide annual health assessments and to offer or refer women for recommended services such as screening, counseling, and preventive care.^48^

Routine outpatient care aims to optimize a woman's health before the onset of pregnancy and ensure timely delivery of prenatal care during pregnancy, thereby reducing the risks for pregnancy complications and hence for both maternal and infant mortality.^16,49^ However, it is important to note that in this study only hospital-based outpatient care was examined. It is unclear whether there were any differences in primary care utilization outside of hospital-based settings between women residing in rural and urban areas.

Overall, this study found some rural–urban differences in health care use among women of reproductive age. Rural–urban differences in health care use, however, were not homogeneous across insurance status groups and further exploration into findings is warranted to better understand their implications. For this population at their peak fertility, high-quality prenatal and preventive care provision aligns well with the concept of value-based care, whereby efficiencies in providing safe and appropriate health care services in outpatient settings ultimately makes populations healthier, patients more satisfied, and prevents the need for more costly emergency care and hospitalizations.^50^ Many recommended preventive health services, including those listed in the HRSA-supported Women's Preventive Services Guidelines, are available to most women with nongrandfathered health insurance plans without having to pay a co-payment, co-insurance, or a deductible.^51^ However, lack of insurance coverage may be a barrier to receiving such care, and particularly so in rural areas.^16,41^

This study is subject to several limitations, including the need to exclude a number of MEPS-HC respondents (18,318) owing to missing values in the individual- and county-level variables, potentially reducing the representativeness of the sample or causing bias in the estimation of the parameters (Appendix Table A1). To this end, those with public health insurance and the uninsured may be represented at somewhat higher levels in the study population than they are in the U.S. population as a whole.^52^ Furthermore, this study examined 10 years of data; however, the most recent year of data available at the time of our analysis (2015) is only 1 year after the major insurance expansions that occurred as a result of the Affordable Care Act (ACA). The ACA affected access to care for women of reproductive age, and it may be wise in the future to perform an updated analysis as more recent data becomes available for study. In addition, as described previously, inherent differences in the values and health care expectations for rural residents may affect their responses to MEPS survey questions around care access and whether rural women seek health care services.^11,53^ For this study, the data set was purposefully restricted to women of reproductive age, not specifically to pregnant women or those hospitalized for maternal health care reasons. The results still have relevance for the study of severe maternal morbidity and mortality in rural areas; however, the findings in this study should not be interpreted as being directly related to maternal health care delivery. Future research could explore the broad array of other issues requiring action for reducing severe maternal morbidity and mortality in rural areas.^54^ Finally, Medicaid expansion and the individual mandate, resulting from the passage of the ACA, may have had effects on the estimations in this study of rural–urban disparities in access to care or medical costs. For example, earlier studies suggest that Medicaid expansion may have both increased insurance coverage for women of reproductive age and reduced their likelihood of experiencing cost barriers to care access.^55^ Our findings about the rural–urban disparities in hospital visits among uninsured individuals might be underestimated given that rural–urban disparities in Medicaid coverage decreased after implementation of the ACA.^56^

## Conclusions

The findings of this study, suggesting variable hospital-based resource use between women residing in rural and urban areas by their insurance status, has relevance for rural population health. Historically, rural women as a population have had poorer overall health and have faced higher rates of severe maternal morbidity and mortality than their urban counterparts. The results presented here highlight the necessity for further research and policy interventions for rural women. Ensuring that rural women of reproductive age are able to access high-quality care both in the outpatient setting and at hospitals is particularly relevant for women experiencing high-risk pregnancies and deliveries and for those with public sources of or no health insurance.

## Disclaimer

Owing to the use of restricted variables, all data analyses in this article were conducted at the Center for Financing, Access, and Cost Trends at the Agency for Healthcare Research and Quality Data Center. The views expressed in this publication are solely the opinions of the authors and do not necessarily reflect the official policies of U.S. Department of Health and Human Services, Health Resources and Services Administration, or the Agency for Healthcare Research and Quality, nor does mention of the department or agency names imply endorsement by the U.S. Government.

## Abbreviations Used

ACA: Affordable Care Act
AHRF: Area Health Resource Files
AHRQ: Healthcare Research and Quality
CDC: Centers for Disease Control and Prevention
CI: confidence interval
ED: emergency department
HRSA: Health Resources and Services Administration
HRSA-OHE: Health Resources and Services Administration—Office of Health Equity
MEPS: Medical Expenditure Panel Survey
ORISE: Oak Ridge Institute for Science and Education
RUCA: Rural-Urban Commuting Area
SCHIP: State Children's Health Insurance Program

## Appendix

**Appendix Table A1.** Determination of the Study Sample

	Sample size
Study sample	
Observations (MEPS 2006**-**2015)	349,405
Restricted to female	182,281
Restricted to age 18 to 44	66,432
Restricted owing to missing data—final sample size	48,114
No. of missing values in the restricted study sample^a^	
Rural/urban	12,543
Education	653
Married	28
Family size	1,779
Activity limitation	2,700
Employment status	1,262
Poor or fair health status	2,091
Poor or fair mental health status	2,126
Pregnancy status	32
Chronic disease	215

^a^ Data were missing for rural status and/or 1+ covariates in some MEPS respondents, making the total number of excluded participants 18,318. Although missing values in the study sample were excluded from the main analysis, respondents outside our study sample were still used for weighting.

MEPS, Medical Expenditure Panel Survey.
